# Clinically derived 12-factor structure and confirmatory factor analysis of the neurodevelopmental parent report for outcome monitoring

**DOI:** 10.3389/fpsyt.2023.1243467

**Published:** 2023-08-30

**Authors:** Nicole T. Baumer, Katherine G. Pawlowski, Joseph L. Amaral, Bo Zhang, Georgios Sideridis, April R. Levin

**Affiliations:** ^1^Division of Developmental Medicine, Boston Children’s Hospital, Boston, MA, United States; ^2^Department of Neurology, Boston Children’s Hospital, Boston, MA, United States; ^3^Harvard Medical School, Boston, MA, United States; ^4^Department of Neurology, The Medical College of Wisconsin, Milwaukee, WI, United States; ^5^Biostatistics and Research Design Center, Institutional Centers for Clinical and Translational Research, Boston Children’s Hospital, Boston, MA, United States

**Keywords:** autism spectrum disorder (ASD), ASD-PROM, ND-PROM, confirmatory factor analysis, parent reported outcome measures, developmental questionnaire

## Abstract

Autism spectrum disorder (ASD) is a neurodevelopmental disorder characterized by social communication and social interaction impairments accompanied by restrictive and repetitive behaviors or interests. Co-occurring conditions may greatly impact overall functioning and intervention needs, and contribute to individual variability and etiologic subtypes. Clinical care of individuals with ASD requires gathering a breadth of information across multiple domains. The neurodevelopmental parent report for outcome monitoring (ND-PROM) was developed to assess symptoms across core features of ASD as well as frequent concerns and comorbidities. The current study expands upon the initially reported psychometric properties of the ND-PROM and evaluates a proposed a clinically derived 12-factor structure of the ND-PROM.

**Methods and procedures:**

The ND-PROM was completed for 246 children with ASD ands tested using confirmatory factor analysis (CFA) and measurement invariance based on sex.

**Outcomes and results:**

A 12-factor correlated structure was found (expressive language, receptive language, nonverbal communication, social emotional understanding, social interaction, independent play, adaptive/toileting skills, restrictive and repetitive behaviors and interests, sensory processes, challenging behaviors, impulse/ADHD, and mental health), which did not vary by sex.

**Conclusions and implications:**

The ND-PROM captures a range of distinct aspects of developmental and behavioral functioning in ASD that can be used to track independent functioning across domains.

## 1. Introduction

Autism spectrum disorder (ASD) is a neurodevelopmental disorder characterized by core impairments in social communication and social interaction accompanied by restrictive and repetitive behaviors or interests (1). ASD impacts individuals across almost every aspect of their lives. The manifestation and severity of core features is quite variable among individuals with ASD, as are cognitive and language abilities, contributing to the significant heterogeneity observed within the ASD population (2). ASD is commonly associated with co-occurring conditions such as depression, anxiety, attention-deficit/hyperactivity disorder, epilepsy, feeding, or sleep problems (3). These co-occurring conditions may greatly impact overall functioning and intervention needs, and contribute to individual variability and etiologic subtypes. Thus, clinical care of individuals with ASD requires that clinicians gather a breadth of information across multiple domains. The neurodevelopmental parent report for outcome monitoring (ASD-ND-PROM) (4) is a caregiver questionnaire that was developed to monitor core ASD symptomatology, as well as the multitude of concerns and comorbidities that may occur in ASD and would need to be addressed during clinical visits, such as communication, social and adaptive skills, repetitive behavior, attention, mood, and maladaptive behavior. Previously called the ASD-PROM, the name of this questionnaire has been updated to reflect the broader clinical populations with overlapping symptomatology, in which this monitoring tool may be useful.

While several parent report instruments are available for screening and diagnosis of core symptoms associated with ASD [e.g., Social Responsiveness Scale, Second Edition (5) and the Gilliam Autism Rating Scale, Third Edition (6)] or symptomatology associated with specific conditions that may co-occur in ASD (e.g., Behavior Assessment System for Children, Third Edition (BASC-3) (7) and the Achenbach System of Empirically Based Assessment (including the Child Behavior Checklist) (8), these scales individually do not capture the full breadth of core symptoms and behaviors seen in the ASD population across early childhood through adolescence, as well as the full range of common comorbidities that can be seen. Additionally, commonly used instruments are generally designed to assess the presence or absence of symptoms to support a categorical diagnosis in a certain age group.

The ND-PROM was developed to address some of these limitations in a single, caregiver-reported measure, by capturing the full breadth of concerns that may need to be addressed during clinical visits for a child with ASD, framed using a strengths-based model of care. The ND-PROM contains 128 Likert-scale items that were compiled by a multidisciplinary team of expert clinicians (including experts in autism, sleep, feeding, toileting, language, communication, and behavior) to facilitate their monitoring of autism patients, as well as those with other neurodevelopmental conditions. Responders indicate the frequency of each assessed developmental skill and behavior on a 5-point Likert scale (never, rarely, sometimes, often, always). It is designed for use with children between the ages of 2 and 20 years and a wide range of developmental levels, containing questions ranging from beginning developmental skills expected in younger children (e.g., “responds when name is called”) to more advanced skills expected in older children (e.g., “has conversations” and “understands non literal language”). The ND-PROM is formatted hierarchically from basic to more advanced skills, and with skip patterns, so that caregivers are not asked questions that are inappropriate for their child. For example, caregivers who indicate that their child is nonverbal are not asked questions about the child’s ability to carry on a conversation; raters of young children are not asked items more relevant for adolescents). Beyond the expressive language scale, items were generally worded to be applicable across a wide range of functioning (e.g., “seems sad” vs. “says they are sad”) (see complete short form PROM with skip pattern in Appendix 1). Finally, the ND-PROM is a tool that is freely available, has both paper and electronic formats, and is available in Spanish and Portuguese. These characteristics set it apart from other validated and reliable instruments that are used in the field.

We previously published the ND-PROM and reported on its clinical utility and initial psychometric properties, including test-retest reliability and convergent validity (4). With regards to clinical utility, parents and clinicians felt that the ND-PROM items described the child’s ability well, and clinicians found that the ND-PROM helped them to provide more patient-centered and efficient care by decreasing time spent taking an extensive history, and increasing time for counseling focused on patient/family priorities. Test-retest reliability assessment revealed high concordance ratings between two administrations of the ND-PROM 2 weeks apart (Pearson *r* correlations 0.95 overall). Convergent validity assessment revealed high correlation of the ND-PROM with the Vineland Adaptive Behavior Scales, Second Edition Parent/Caregiver Rating Form (4, 9).

Following development of the ND-PROM, and original publication of the tool, the ND-PROM clinical research team further refined the measure. The original ND-PROM listed questions under four headings meant to orient patients and clinicians to question type: (1) communication and social skills (51 items), (2) adaptive skills (3 items), (3) behavioral functioning (53 items), and (4) sleep (14 items). These headings did not provide clinicians with the ability to easily track developmental and behavioral functioning according to clinically relevant areas that may change over time independent of each other. For example, a child may show great improvement in repetitive behaviors, but may develop symptoms consistent with co-occurring ADHD/impulsivity issues over time. Shifting to having independent scores to track separate areas of functioning provides more clinical utility.

The current study expands upon the initially reported psychometric properties of the ND-PROM and proposes a clinically derived 12-factor structure of the ND-PROM. We performed confirmatory factor analysis conducted based upon the 12-factor structure and assess internal structure validity of the ND-PROM.

## 2. Methods

### 2.1. Participants and procedures

The ND-PROM was completed by parent or caregiver for 246 individuals with an established clinical diagnosis of ASD provided by an autism specialist at a specialty pediatric referral hospital from 2016 to 2018 (herein referred to as “participants”). Clinical diagnosis was established by autism specialists who regularly assess for and treat ASD within an established Autism Spectrum Center with standardized diagnostic approaches using DSM-5 criteria (1). These clinicians included developmental behavioral pediatricians, pediatric psychologists, and child neurologists or neurodevelopmental disability specialists. Participants were drawn from specialty clinics in Developmental Medicine and Neurology, where they were followed by these specialty clinicians for care related to their ASD diagnosis. This study was approved by the institution’s Institutional Review Board. Sixty-two of the 246 participants participated in the initial study (4). The remaining 184 participants were retrospectively identified from a sample of patients who had completed the ND-PROM for clinical purposes. Parents or caregivers completed the ND-PROM electronically, using a web-based system in which parents received automated prescheduled emails with secure links to complete the questionnaire online.

### 2.2. Study instrument: clinically derived 12-factor structure

In the present study, the ND-PROM items were delineated into the following 12 clinically-determined factors: (1) expressive language, (2) receptive language, (3) nonverbal communication, (4) social emotional understanding, (5) social interaction, (6) independent play, (7) adaptive/toileting skills, (8) restricted and repetitive behaviors and interests, (9) sensory processes, (10) challenging behaviors, (11) impulse/ADHD, (12) mental health. questions pertaining to sleep, epilepsy, and feeding problems were not included in the proposed factor structure, as these items were expected to be independent questions, and not related to other items. The 12-factor model was tested and revised iteratively, with inclusion and exclusion of various items to establish the best fit model and final factor structure. Iterations involved tests of individual items using Lagrange and Wald tests and their effect on global model fit.

### 2.3. Confirmatory factor analysis of ND-PROM

A confirmatory factor analysis (CFA) model was used to evaluate how well our data fit the clinically-determined 12-factor structure of the ND-PROM. Both global and local CFA models were utilized to evaluate the dimension and underlying structure of the ND-PROM (internal structure validity). The global model involved a 12-correlated factor structure using the weighted least squares mean and variance adjusted (WLSMV) estimator, as is appropriate for ordinal data that likely do not meet the normality assumption. This model was contrasted to a unidimensional structure, justified on the grounds that some domains were highly correlated and thus, a global functioning domain was a plausible hypothesis (10). Because the two models were nested, a chi-square difference test was utilized to conclude the preferred model. These models were run using Mplus (version 8.10). Local analyses (i.e., per domain) were conducted using the graded response model (GRM) (11), and with the use of Mplus and IRTPro.

As internal consistency reliability provides evidence for internal structure validity, we also evaluated the internal consistency reliability of the 12-factor structure of the ND-PROM using Omega coefficient (as opposed to the alpha coefficient), because of the following previously described shortcomings of alpha coefficient: (a) its positive bias with large instruments, (b) its conservatism by being a low bound estimate of the true reliability, and, (c) its unsuitability for non-tau equivalent instruments, as it aggregates item-latent variable correlations rather than including the specific contributions and respective errors of each item to the estimation of reliability (12).

We also assessed measurement invariance across sex, to test whether the factor structure is similar across males and females. This ensures that observed mean differences across groups reflect true differences, rather than differences that are reflective of differentially functioning instruments (13, 14). This is important given the understanding of sex as a biological construct, and the debated potential differences in ASD presentation across sexes (15–17). As a minimum of three levels of invariance are required prior to conducting meaningful tests of differences, we assessed configural, metric, and scalar invariance across the two groups (males and females) (18). First, we conducted analyses of configural invariance to assess whether the number of factors and pattern of loadings were the same for both males and females. Next, we assessed metric invariance to determine whether the actual magnitude of the loadings were the same across males and females for each respective item. Finally, we assessed scalar invariance, which imposes the same constraints as configural and metric invariance, but has the added constraint that the thresholds are equated across the two groups, which is required for comparison of latent means. Violation of scalar invariance is often termed differential item functioning (DIF). Not meeting scalar invariance assumptions precludes comparisons of means between groups; therefore, for situations in which scalar invariance is not met, there are two possible approaches: (a) partial measurement invariance (19) and (b) satisfaction of measurement invariance using an approximate protocol (20). In the present study, we adopted an approximate invariance protocol when necessary (21, 22). This posits that minor deviations between estimated parameters reflect an amount of random error that we could simply ignore as being too small to be harmful. Thus, Bayesian priors are posited on variances only, and not on means, because groups should vary around the population estimate, but should not differ in meaningful ways. In other words, some variability around zero mean estimates should be allowed to provide for “wiggle room” (23) so that the equivalence of the measuring instrument is justified, and between-group inferences in level are consequently valid. This prior variance estimate defines a level of variability in the parameters between groups that is sufficiently small so that parameters are considered invariant assuming that an optimal prior variance estimate is selected. If approximate invariance is satisfied, then latent factor means are not contaminated by measurement error due to between groups differences, and inferences with regard to level can be further conducted.

## 3. Results

### 3.1. Sociodemographics

Table 1 shows descriptive statistics of the participants. As shown in the table, the majority of participants were males (83.3%), white (77.3%) and non-Hispanic (93.6%). Furthermore, a little over 50% of caregiver responders had completed a college degree. The median age of participants was 8.8 years (IQR 6.4–11.9); median age of participants who did not yet communicate was 5.6 years (IQR 3.1–8.9); median age of those who used full sentences to communicate was 9.60 (8.3–11.7).

**Table 1:** Demographic characteristics of study participants.

	Participants
Age, median (IQR)	8.8 (6.4–11.9)

Sex	*n* (%)
Male	205 (83.3)
Female	41 (16.7)

Race (*n* = 200)	*n* (%)
American Indian/Alaska native	1 (0.6)
Asian	9 (5.2)
Black/African American	9 (5.2)
White	133 (77.3)
Other	20 (11.6)
Unknown/not reported	28 (6.4)

Ethnicity (*n* = 156)	*n* (%)
Hispanic	10 (6.4)
Non-Hispanic	146 (93.6)
Unknown/not reported	44 (10.0)

Responder education (*n* = 178)	*n* (%)
Did not complete high school	5 (2.8)
Completed high school but not college	17 (9.4)
Completed college or above	92 (50.8)
Unknown/not reported	67 (37)

Primary communication type	Median age (IQR)
Spoken language (*n* = 211)	9.39 (7.82–11.52)
Sign language (*n* = 9)	2.59 (2.30–5.57)
Picture communication system (e.g., PECS) (*n* = 12)	5.81 (4.08–7.52)
Electronic communication system (e.g., Dynavox, iPad) (*n* = 6)	9.16 (7.07–11.87)

Maximum length of communicative units	Median age (IQR)
Does not yet communicate (*n* = 10)	5.62 (3.07–8.87)
Uses one word/picture/sign at a time (*n* = 20)	4.41 (3.70–7.38)
Uses to words/pictures/signs at a time (*n* = 14)	5.68 (3.36–9.31)
Uses three words/pictures/signs at a time (*n* = 26)	8.20 (6.23–10.97)
Uses full sentences (*n* = 176)	9.60 (8.27–11.72)

Sociodemographic and responder education level for *n* = 246 participants. Median and interquartile range (IQR) is shown for age, primary communication type, and maximum length of communicative units; all other factors are presented with n and percent reporting. Race, ethnicity, and responder education were taken from the medical record and were not available for all participants.

### 3.2. 12-factor structure of ND-PROM and confirmatory factor analysis

Table 2 shows the 12-factor structure and the question items contained within each factor, as well as questionnaire items that were excluded from the factor structure. The factors were: expressive language (10 items), receptive language (6 items), nonverbal communication (6 items), social emotional understanding (8 items), social interaction (12 items), and independent play (3 items), adaptive/toileting skills (6 items), restricted and repetitive behaviors and interests (15 items), sensory processes (8 items), challenging behaviors (6 items), impulse/ADHD (4 items), and mental health (10 items).

**Table 2:** Standardized factor loadings from a confirmatory factor analysis model for the 12 latent variables of the ND-PROM.

Factor number	Factor	Items	Factor loading
1	Expressive language	Indicates yes/no	0.798
		Uses names of objects	0.918
		Requests/asks for things	0.821
		Makes comments	0.929
		Tells others what to do	0.855
		Asks “Why” questions	0.875
		Tells you about an event that happened in the past	0.903
		Has conversations	0.919
		Communicates spontaneously (initiates)	0.812
		Pronounces words correctly	0.767
2	Receptive language	Understands when told yes/no	0.705
		Understands 1 step directions	0.895
		Understands 2 step directions	0.874
		Understands if/then	0.858
		Understands non-literal language	0.937
		Responds when name is called	0.708
3	Nonverbal communication	Points to indicate wants	0.824
		Points to share interest when not requesting	0.856
		Gestures	0.815
		Makes appropriate eye contact	0.499
		Uses facial expressions to show feeling	0.634
		Combines eye contact, gestures, facial expressions appropriately	0.844
4	Social emotional understanding	Distinguishes friendly teasing from bullying	0.884
		Recognizes emotions of others	0.701
		Demonstrates sportsmanship	0.864
		Identifies own feelings	0.71
		Understands others may have different point of view	0.823
		Shows remorse (being sorry)	0.744
		Handles criticism well	0.667
		Offers comfort to others	0.699
5	Social interaction	Appropriately gets someone’s attention to start/end interaction	0.747
		Understands personal space	0.737
		Seems interested in interacting with children he/she knows	0.683
		Responds appropriately to greetings from children he/she knows	0.779
		Plays with classmate with help	0.582
		Plays with classmate without help	0.849
		Plays in group of classmates without help	0.84
		Imitates or copies others to learn	0.536
		Plays simple social games	0.704
		Plays cooperative games/taking turns and following rules	0.854
		Attempts to contact familiar children outside of school	0.774
		Understands social relationships	0.93
6	Independent play	Engages in simple pretend play	0.674
		Acts out scene (scripted play)	0.757
		Pretends to be superhero or other character	0.892
7	Adaptive/toileting skills	Potty trained day	0.946
		Cleans/wipes	0.912
		Dresses independently	0.925
		Smears/plays with stool/urine (R)	0.193
		Toilets inappropriate places (R)	0.733
		Holds back stool (R)	0.533
8	Restricted and repetitive behaviors and interests	Focuses on unusual interests that interfere	0.549
		Focuses on intense interests that interfere	0.542
		Repetitive movements	0.656
		Simple repetitive activities	0.751
		Focuses on parts of objects	0.923
		Compulsions/rituals	0.474
		Avoids/upset about new places/people	0.586
		Easily upset with changes in routine	0.675
		Difficulty with transition	0.628
		Needs you to change your behavior to avoid becoming upset	0.53
		Speaks in unusual tone of voice	0.506
		Repeats meaningless sounds	0.806
		Echoes other people	0.482
		Repeats phrases from TV/movies	0.403
		Perseverates or gets stuck	0.282
9	Sensory processes	Peers out of corner of eyes	0.534
		Craves deep pressure	0.489
		Upset by noises	0.277
		Puts things into mouth that are not food	0.624
		Avoids touching certain things	0.543
		High tolerance for pain	0.377
		Holds or packs food in mouth	0.157
		Eats limited variety of foods	0.612
10	Challenging behaviors	Physically aggressive toward self	0.757
		Physically aggressive towards others	0.686
		Expresses thoughts of wanting to hurt others	0.465
		Destroys or breaks things when upset	0.621
		Temper tantrums or meltdowns	0.782
		Interrupts when others are speaking	0.12
11	Impulse/ADHD	Runs away	1
		Easily distracted, difficulty paying attention	0.457
		Hyperactive	0.643
		Impulsive, acts without thinking	0.699
12	Mental health	Expresses self-harm or suicide	0.67
		Victim of bullying	0.544
		Worries too much	0.634
		Picks at skin or nails	0.501
		Seems sad	0.695
		Easily frustrated	0.801
		Sudden changes in mood	0.808
		Sees things not there	0.676
		Hears things not there	0.744
		Decreased or flattened emotions	0.519

Assessment of internal structure validity revealed that the items within each of the 12 factors performed well in relation to each other and were consistent with the clinically derived headings of items expected to be related to each other. Global model fit was evaluated by testing a 12-factor correlated structure using an item factor analysis (IFA) treating the responses as ordinal and adjusting lack of normality using the WLSMV estimator. Results after adjusting for sample size using the Bartlett correction^1^ indicated acceptable model fit as evidenced by an RMSEA = 6% and a comparative fit index (CFI) = 0.901. Based on Hu and Bentler (24) when RMSEA estimates are equal to or below 0.06 and a descriptive fit index is greater than 0.900, this is evidence for acceptable model fit. This 12-construct structure was contrasted to a unidimensional construct to rule out the hypothesis that there was a single “general” factor that captured all behaviors. After contrasting the two nested models using a chi-square difference test, results indicated preference for the 12-factor correlated model [difference chi-square (66) = 2555.107, *p* < 0.001].

Further assessment of each factor was completed using a series of GRM models to specifically test for local misfit, in light of the strong resemblance between CFA and item response models. GRM models were applied to each factor of the ND-PROM in order to test for subscale adequacy through examining item fit, correlated residuals, and internal consistency reliability estimates. As shown in Table 3, using unstandardized residuals (i.e., RMSEA), all domains except social interaction and adaptive skills were acceptable. The relatively large RMSEA of social interaction and adaptive skills is likely explained by the complexity of the construct, and the small number of items, respectively. However, the strength of the model using other indicators, in addition to RMSEA, shows the strength of the model overall to explaining the observed relationships between items and the latent variables. Furthermore, all estimates of internal consistency reliability were within an acceptable range (0.72–0.94) and local dependency was almost non-existent, ranging between 0% and 17.8%. There were a total of 386 residual correlations from which 32 were significantly different from zero. This amount reflects 8.3% of the total number of tests which is just above the nominal level of significance (i.e., what is expected by chance alone). Similarly, item fit statistics in the form of chi-square tests were mostly non-significant and reflected an average of 13.8%. These percentages reflect relatively small deviations from expectations because these expectations are based on a properly powered study of specifically the chi-square test. However, when estimating the power of the test given the observed number of response patterns (i.e., *df* = 40), the required number of participants was *n* = 112. Thus, if we had *n* = 112 we would expect 5% of the significant tests to represent what would be expected by chance alone. Our numbers, however, were twice as much and thus, with a sample size of *n* = 246 the chi-square tests is heavily over-powered. Thus, any deviations from model expectations would likely result in significant results, pointing to the presence of misfit. This is why, given excessive power for this specific analysis, we conclude that our observed significant tests of 13.8% reflect minuscule deviations of the observed response patterns to those of the Guttman pattern (i.e., the expectation that an average skilled person would be highly successful on the easy items/behaviors, less successful on items/behaviors of medium difficulty, and mostly unsuccessful on difficult items/behaviors). The high internal consistency demonstrates high reliability of the subscales, an indication that the individual questionnaire items within each factor appropriately reflect functioning within that clinical domain, and that the individual factors measure unique aspects of functioning in individuals with ASD.

**Table 3:** Subscale model fit of ND-PROM using the graded response model.

ND-PROM subscales	M2	RMSEA	Marginal rel.	Item fit *S*-chi-square	Local dependency
					(*N*/%)
F1: Expressive language	1485.55^***^	0.05	0.94	2/10	0/45 (0)
F2: Receptive language	281.61^***^	0.03	0.87	0/6	0/15 (0)
F3: Nonverbal communication	881.47^***^	0.08	0.86	0/6	0/15 (0)
F4: Social emotional understanding	1302.01	0.07	0.89	0/8	5/28 (17.8)
F5: Social interaction	11526.22^***^	0.15	0.93	1/12	11/66 (16.7)
F6: Independent play	119.61	0.01	0.82	0/3	0/3 (0)
F7: Adaptive/toileting skills	2050.91^***^	0.14	0.78	0/6	1/15 (6.7)
F8: Restricted and repetitive behaviors and interests	4345.48^***^	0.06	0.89	2/15	11/105 (10.5)
F9: Sensory processes	672.51^***^	0.03	0.72	5/8	0/28 (0)
F10: Challenging behaviors	323.38^***^	0.03	0.79	2/6	0/15 (0)
F11: Impulse/ADHD	230.85^***^	0.06	0.80	1/4	2/6 (33.3)
F12: Mental health	913.31^***^	0.03	0.85	0/10	2/45 (0.4)

M2 inferential test and the unstandardized residuals (root mean squared error of approximation, RMSEA) are reported. *p*-values are adjusted using the Benjamini–Hochberg correction using a false discovery rate equal to 5% for a two-tailed test. Local dependency was evaluated at an alpha level of 0.01 (critical value = 6.635 using 1 degree of freedom).

^***^Designation is significant at *p* < 0.001.

Measurement invariance of the ND-PROM factor structure was assessed across sex, to ensure valid comparisons between males and females at their mean level. Table 4 displays the results from testing the measurement invariance of the measure across sex. As shown in the table, two comparisons were of interest, the difference between metric and configural models and the difference between metric and scalar models. Both are prerequisite to conducting unbiased comparisons between groups in level for these factors. The lack of measurement invariance was observed in two comparisons, the slopes of factor 8 (restricted and repetitive behaviors and interests) and the intercepts of factor 12 (mental health). Follow-up analyses on differential slopes and/or intercepts were conducted. For factor 8, restricted and repetitive behaviors and interests, there were significant differences in the slopes of items 5 and 15, which, after applying an approximate invariance protocol, became non-significant.

**Table 4:** Measurement invariance of ND-PROM across sex.

ASD-PROM factors	Measurement invariance	Chi-square	D.F.	*p*-value
F1: Expressive language	Metric vs. configural	8.422	9	0.492
	Scalar vs. metric	2.619	9	0.978
F2: Receptive language	Metric vs. configural	1.984	5	0.851
	Scalar vs. metric	8.049	5	0.154
F3: Nonverbal communication	Metric vs. configural	2.544	5	0.769
	Scalar vs. metric	6.685	5	0.245
F4: Social emotional understanding	Metric vs. configural	3.073	7	0.878
	Scalar vs. metric	7.114	7	0.417
F5: Social interaction	Metric vs. configural	10.443	11	0.491
	Scalar vs. metric	5.378	11	0.912
F6: Independent play	Metric vs. configural	1.77	2	0.413
	Scalar vs. metric	1.08	2	0.583
F7: Adaptive/toileting skills	Metric vs. configural	6.117	5	0.295
	Scalar vs. metric	7.654	5	0.176
F8: Restricted and repetitive behaviors and interests	Metric vs. configural	50.591	14	<0.001^**^
	Scalar vs. metric	13.117	14	0.517
F9: Sensory processes	Metric vs. configural	12.121	7	0.097
	Scalar vs. metric	8.993	7	0.253
F10: Challenging behaviors	Metric vs. configural	3.071	5	0.689
	Scalar vs. metric	9.597	5	0.088
F11: Impulse/ADHD	Metric vs. configural	3.542	3	0.315
	Scalar vs. metric	1.004	3	0.800
F12: Mental health	Metric vs. configural	5.300	9	0.807
	Scalar vs. metric	18.157	9	0.033^*^

^*^ = significant at *p* < 0.05. ^***^ = significant at *p* < 0.001. D.F., degrees of freedom.

**Figure 1:**
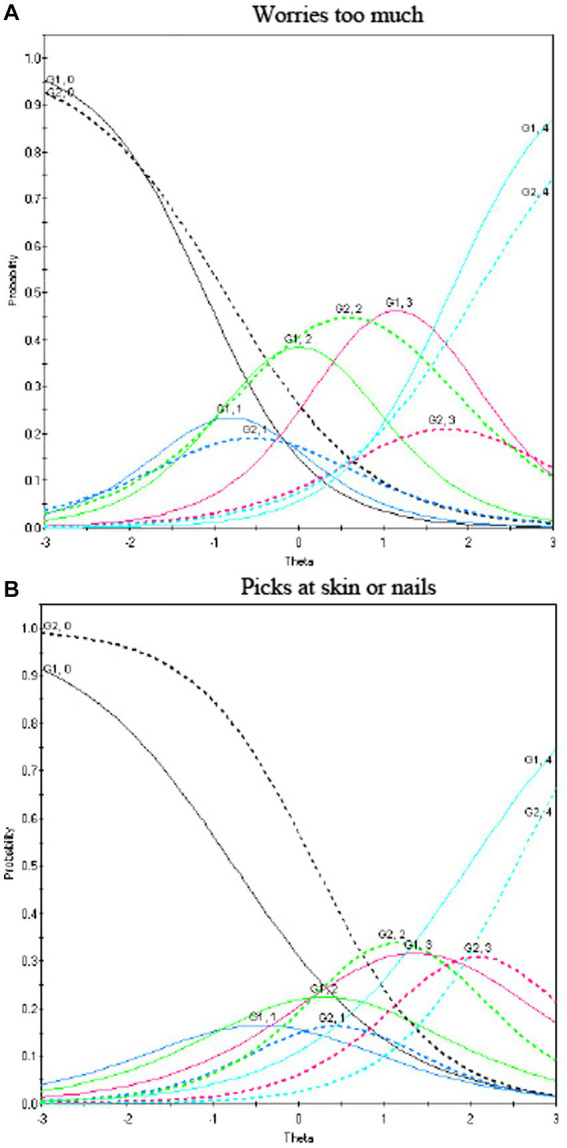
Differential item functioning for mental health disorder symptoms of the ND-PROM. Differences in responses to the items “worries too much” **(A)** and “picks at skin or nails” **(B)** from the mental health factor as a function of sex. Lines represent category curves for response options 0 = never, 1 = rarely, 2 = sometimes, 3 = often, and 4 = always. Solid lines represent males and dashed lines females. The horizontal axis (termed theta) represents levels of the latent trait, i.e., worrying too much (low to average to high scores, from left to right). The same is also true of the item “pick at skin or nails.”

For the mental health factor, results pointed to the difference between males and females on two items. As shown in Figure 1, males were more likely to have “often” responses endorsed, compared to females, on both the “worries too much” (Figure 1A) and “picks at skin or nails” (Figure 1B) items. Furthermore, in order to test the hypothesis that the levels of measurement non-invariance were not prohibitive of conducted mean comparisons a Bayesian approximate measurement invariance protocol was applied as discussed above. Results, after allowing a non-negligible variance between groups on the intercepts at a level of 0.01, indicated no significant differences between males and females on those terms. The levels of non-invariance were classified as “negligible” and justify valid tests of means across males and females. Thus, collectively these results suggest that comparisons between latent means across sex can be conducted as these point estimates proved to be bias-free.

## 4. Discussion

In this study we expand upon initially reported psychometric properties of the PROM, by conducting confirmatory factor analysis of a clinically proposed 12-factor structure and evaluating measurement invariance across sex. Our findings suggest that 12 clinically derived factors, which represent independent domains of functioning in ASD, were well-supported and show that the ND-PROM captures a range of distinct aspects of developmental and behavioral functioning in ASD, and that each factor contains multiple correlated question items which can be used to independently track functioning across domains.

Factor analysis indicated a good statistical fit of a model with 12 clinically-meaningful factors. The best fit was a multi-factor structure solution that contained factors related to DSM-5 diagnostic criteria for ASD as well as factors that address common comorbidities such as maladaptive and aggressive behaviors, impulse/ADHD symptoms, mental health problems, and adaptive skill deficits commonly seen in ASD. The factor structure included six factors directly related to core ASD symptomatology, which map closely onto DSM-5 Diagnostic Criteria for ASD: Nonverbal Communication (DSM 5 A2), Social Emotional Understanding (DSM 5 A3), Social Interaction (DSM 5 A1 and A3), Independent Play (DSM 5 A3), Restrictive and Repetitive Behaviors and Interests (DSM 5 B1, B2, B3), and Sensory Processes (DSM 5 B4) (1). The factor structure also included six factors that delineate associated skills, symptoms, and conditions that are within domains not directly related to core ASD symptomatology: expressive language, receptive language, adaptive skills/toileting, challenging behaviors, impulse/ADHD, mental health. Certain comorbidities such as sleep problems, feeding problems, and epilepsy were not included within the factor structure of the ND-PROM, though items in each of these areas were included in the questionnaire, as they are still clinically meaningful symptoms to track over time and have utility as stand-alone questions on the ND-PROM.

Previous studies have found multiple factors using scales commonly used in ASD. For example, on the Autism Diagnostic Observation Schedule (ADOS), which specifically assesses core ASD symptoms, a three factor model consisting of “repetitive behaviors” and two separate social-communication factors (“basic social-communication” and “interaction quality”) were found (25). Other researchers have found subdomains within the repetitive and restrictive behaviors domain on the repetitive behavior scale-revised (RBS-R) (“stereotypic behavior,” “self-injurious behaviors,” “restricted interests,” “compulsive behavior,” “ritualistic/sameness behavior”) (26–28). Prior research has also explored measurement of behavior problems in ASD using the Aberrant Behavior Checklist (ABC) and have identified subgroups based on problem behaviors and psychopathology in ASD not related to core ASD symptomatology (29). In our study, as the ND-PROM contains multiple items intended to capture a breadth of relevant domains, and is assessed in a population of varying ages and abilities, it is not unexpected that a multi-factor solution fits best.

At its current stage of development, the main purpose of the ND-PROM is to provide a snapshot of functioning in multiple domains that covers both core ASD symptomology and co-occurring conditions among individuals with ASD. However, it should be noted that the ND-PROM is not norm-referenced, and it is not possible to infer the degree to which scores correlate to absolute symptom levels or levels of functioning. However, given that ND-PROM is not designed to be a diagnostic tool, but rather to be used in conjunction with conversations between a clinician and family, if concern is raised for a particular issue (e.g., depression), further assessment, in some cases utilizing existing norm-referenced instruments, can be conducted. Through ongoing collection of longitudinal data, future studies can assess the ability of the ND-PROM to document change and response to treatment over time, to identify phenotypically defined subgroups within the ASD population, and to assess the utility and psychometric properties of the ND-PROM in other neurodevelopmental/neurogenetic populations (e.g., down syndrome). In the future, scalability of the ND-PROM could be increased by developing a brief form employing computerized adaptive testing (CAT).

Several limitations of the study should be considered. The first is the generalizability of initial findings to the broader autism population. Participants were patients of specialty clinics at a pediatric referral center, and not recruited from the community, thus it is possible that study sample demographic characteristics are not representative of the full spectrum of individuals with ASD, and may have had more behavioral or co-occurring medical challenges than their peers in the community. Participants tended to identify as White, non-Hispanic. The ND-PROM has since been translated into Spanish and Portuguese, and future studies will seek to include a more diverse population. Information collected is caregiver-reported and may not match evaluation by a trained clinician. Although the ND-PROM can be administered on paper, in this study it was administered electronically, and therefore naturally excluded individuals without access to email and technology. Additionally, this clinically-derived sample was not subjected to diagnostic confirmation for the purposes of the current study. Instead, we relied on the established diagnosis conferred by a specialty clinician. Information about the severity of ASD symptoms or comorbid conditions was not available for the current study sample, and the degree to which communication or developmental differences may have impacted item loading and the overall factor structure was not explicitly explored. It should be noted that the population studied did include those with a wide range of communication and cognitive abilities, although standardized data in these areas was not available for analysis. As additional data is collected, future studies should evaluate the performance of the ND-PROM within specific age or developmental ranges, and can be used to compare functioning across different groups, such as males vs. females, or those with ASD vs. those with other neurodevelopmental conditions. Despite these limitations, our findings suggest that the ND-PROM has good potential value for assessing key independent domains of functioning in individuals with ASD, including those associated with core ASD symptomatology, as well as other relevant areas including language skills, adaptive skills, and symptoms related to co-occurring neurodevelopmental, behavioral, and mental health conditions.

## 5. Conclusion

The ND-PROM is a clinically useful tool that assesses a breadth of symptoms in ASD including core ASD symptoms as well as potential co-occurring medical, developmental, and behavioral concerns. A 12-factor model is well supported by confirmatory factor analysis, convergent and divergent validity, and measurement invariance across sex, suggesting strong construct validity of the ND-PROM in the ASD population. Findings suggest that the ND-PROM has good potential value for independently assessing key functional domains, which can identify targeted areas for intervention.

## Data availability statement

The raw data supporting the conclusions of this article will be made available by the authors, without undue reservation.

## Ethics statement

The studies involving humans were approved by Boston Children’s Hospital Institutional Review Board. The studies were conducted in accordance with the local legislation and institutional requirements. Written informed consent or a waiver of informed consent was provided for the participants in this study.

## Author contributions

NB prepared initial draft of manuscript. GS and BZ performed data analysis. NB, KP, JA, BZ, GS, and AL contributed to study conception and design, led by AL. All authors contributed to the article and approved the submitted version.

## Conflict of interest

The authors declare that the research was conducted in the absence of any commercial or financial relationships that could be construed as a potential conflict of interest.

## Publisher’s note

All claims expressed in this article are solely those of the authors and do not necessarily represent those of their affiliated organizations, or those of the publisher, the editors and the reviewers. Any product that may be evaluated in this article, or claim that may be made by its manufacturer, is not guaranteed or endorsed by the publisher.

## Footnotes

^1^The specific R function developed for this purpose can be found at: https://github.com/GS1968/CFAtools/blob/main/cfagofi1.R.
