# Supplementary material for: Clinically derived 12-factor structure and confirmatory factor analysis of the neurodevelopmental parent report for outcome monitoring
Source: Front Psychiatry. 2023 Aug 30;14:1243467. doi: 10.3389/fpsyt.2023.1243467 (PMC10499167; doi:10.3389/fpsyt.2023.1243467)
Supplement: Supplementary file 1 [file Data_Sheet_1.PDF]

Neurodevelopmental Parent Report for Outcome Monitoring
ND-ASD-PROM Short Form ©

Patient Name:

Date of Birth:

Completed by:

Today's Date:

Please answer all questions as they apply to your child OVER THE LAST 6 MONTHS. All children function differently from very early skills to more complex skills. If there is a skill listed that your child has already mastered independently **without prompting or reminders**, please mark as **Always**. If there is a skill listed beyond your child's developmental level, please mark **Never**. Please fill this form out to the best of your ability and discuss any additional concerns with your physician.
Please fill in ONLY one bubble in each row.

Communication

When your child wants to communicate, how often does he or she use each of the following:

	Never	Rarely	Sometimes	Often	Always
1. Spoken language	◯	◯	◯	◯	◯
2. Sign language	◯	◯	◯	◯	◯
3. Picture communication system (e.g., PECS)	◯	◯	◯	◯	◯
4. An electronic communication device (e.g., iPad, iPhone, Dynavox)	◯	◯	◯	◯	◯

5. What is your child's primary form of communication?

◯ Spoken language
◯ Sign Language
◯ A picture communication system
◯ An electronic communication device

6. When your child communicates, how many words/signs/pictures does your child put together to communicate?

◯ My child does not yet communicate (Note: please skip questions 8-15)
◯ My child uses one word/sign/picture at a time
◯ My child uses two words/signs/pictures at a time
◯ My child uses three words/signs/pictures at a time
◯ My child uses full sentences

© 2023 Boston Children's Hospital. All Rights Reserved.
For more information, contact the PROM Study Team: ASDPROM@childrens.harvard.edu.

Expressive Language

Please answer questions 7-16 based on your child's primary form of communication. How often does he or she do the following appropriately without prompting:

	Never	Rarely	Sometimes	Often	Always
7. Indicates "Yes" or "No"	◯	◯	◯	◯	◯
8. Uses names of objects	◯	◯	◯	◯	◯
9. Requests/asks for things	◯	◯	◯	◯	◯
10. Makes comments (e.g., "Look! Dog!")	◯	◯	◯	◯	◯
11. Tells others what to do	◯	◯	◯	◯	◯
12. Asks "why" questions	◯	◯	◯	◯	◯
13. Tells you about an event that happened in the past	◯	◯	◯	◯	◯
14. Has conversations (e.g. able to maintain back and forth conversation, does not cut off others)	◯	◯	◯	◯	◯
15. Communicates spontaneously (e.g., initiates communication with other people)	◯	◯	◯	◯	◯
16. Pronounces words correctly **(Note: If your child does not use spoken language, please chose Never)**	◯	◯	◯	◯	◯

Receptive Language

Please answer questions 7-15 based on your child's primary form of communication. How often does he or she do the following appropriately without prompting:

	Never	Rarely	Sometimes	Often	Always
17. Understands when told "Yes" or "No"	◯	◯	◯	◯	◯
18. Understands one-step directions (e.g., "Go get your shoes.") **(Note: If Never, please skip to #21)**	◯	◯	◯	◯	◯
19. Understands two-step directions (e.g., "Put the toy away, then go get your shoes.")	◯	◯	◯	◯	◯

© 2023 Boston Children's Hospital. All Rights Reserved.
For more information, contact the PROM Study Team: ASDPROM@childrens.harvard.edu.

Receptive Language

How often does your child do each of the following appropriately:

	Never	Rarely	Sometimes	Often	Always
20. Understands if-then directions (e.g., "If you eat the broccoli, then you can have a cookie.")	○	○	○	○	○
**(Note: If your child is <u>5 years old or younger</u>, please skip to #22)** 21. Understands non literal language such as jokes, sarcasm, or idioms like "He's pulling your leg."	○	○	○	○	○
22. Responds when name is called	○	○	○	○	○

Non-Verbal Communication

How often does your child do each of the following appropriately:

	Never	Rarely	Sometimes	Often	Always
23. Is able to point to indicate what he or she wants	○	○	○	○	○
24. Points to share interest when not requesting (e.g., shows you an airplane in the sky)	○	○	○	○	○
25. Gestures (e.g., nods for yes, waves goodbye)	○	○	○	○	○
26. Makes appropriate eye contact (e.g., looks at you when making a request but does not look too long or make people uncomfortable)	○	○	○	○	○
27. Uses facial expressions to show how he or she is feeling (e.g., smiles when happy, frowns when sad)	○	○	○	○	○
28. Combines eye contact, gestures, and facial expressions appropriately when speaking (e.g., sharing information using both speech and body language to communicate with the listener)	○	○	○	○	○

© 2023 Boston Children's Hospital. All Rights Reserved.
For more information, contact the PROM Study Team: ASDPROM@childrens.harvard.edu.

Social Interaction

How often does your child do each of the following appropriately:

	Never	Rarely	Sometimes	Often	Always
29. Appropriately gets someone's attention to start or end an interaction (e.g., calls name, taps shoulder, or makes eye contact before speaking)	○	○	○	○	○
30. Understands personal space	○	○	○	○	○
31. Seems interested in interacting with children he/she knows	○	○	○	○	○
32. Responds appropriately to greetings from children he/she knows	○	○	○	○	○
33. Plays with a classmate (or other familiar child) **with** help from an adult	○	○	○	○	○
34. Plays with a classmate (or other familiar child) **without** help from an adult	○	○	○	○	○
35. Plays in a group of classmates (or other familiar children) **without** help from an adult	○	○	○	○	○
36. Imitates or copies others as a means of learning	○	○	○	○	○
37. Plays simple social games (e.g., peek-a-boo, tag, hide-and-seek)	○	○	○	○	○
**(Note: If your child is 5 years old or younger, please skip)** 38. Plays cooperative games that require taking turns and following rules (e.g., board games, sports)	○	○	○	○	○
**(Note: if your child is 5 years old or younger, please skip)** 39. Attempts to contact familiar children outside of school	○	○	○	○	○
40. Understands social relationships (e.g. friendship and marriage)	○	○	○	○	○

© 2023 Boston Children's Hospital. All Rights Reserved.
For more information, contact the PROM Study Team: ASDPROM@childrens.harvard.edu.

Social Emotional Understanding

How often does your child do each of the following appropriately:

	Never	Rarely	Sometimes	Often	Always
**(Note: If your child is 5 years old or younger, please skip)** 41. Distinguishes friendly teasing from bullying	○	○	○	○	○
42. Recognizes the emotions of other people	○	○	○	○	○
**(Note: If your child is 5 years old or younger, please skip)** 43. Demonstrates sportsmanship; able to win and lose in a kind and courteous way	○	○	○	○	○
44. Identifies his or her own feelings	○	○	○	○	○
45. Understands that others may have a different point of view from his or her own	○	○	○	○	○
46. Shows remorse (being sorry) for mistakes	○	○	○	○	○
47. Handles criticism well	○	○	○	○	○
48. Offers comfort to others (e.g., gives you a hug if you are sad)	○	○	○	○	○

Independent Play

How How often does your child do each of the following appropriately:

	Never	Rarely	Sometimes	Often	Always
**(Note: If your child is 6 years old or older, please skip to #48)** 49. Engages in simple pretend play (e.g., feeds a doll, pushes a toy car)	○	○	○	○	○
50. Acts out a scene he or she has seen before, such as a scene from a movie ("Scripted play")	○	○	○	○	○
51. Pretends to be a superhero, teacher, or other character ("Novel role play")	○	○	○	○	○

© 2023 Boston Children's Hospital. All Rights Reserved.
For more information, contact the PROM Study Team: ASDPROM@childrens.harvard.edu.

Restricted and Repetitive Behaviors and Interests

To what extent does your child do each of the following:

	Never	Rarely	Sometimes	Often	Always
52. Focuses on **unusual** interests that interfere with daily functioning (e.g., train schedules, interests that most other children wouldn't have)	○	○	○	○	○
53. Focuses on **intense** interests that interfere with daily functioning (e.g., much more interested in dinosaurs than other children are)	○	○	○	○	○
54. Has repetitive movements (e.g., hand flapping, finger wiggling, jumping)	○	○	○	○	○
55. Does simple repetitive activities (e.g., shaking string, ripping paper, turning lights on and off,watching parts of videos over and over again)	○	○	○	○	○
56. Focuses on certain parts of objects (e.g., turns a toy car upside-down to spin the wheels)	○	○	○	○	○
57. Has compulsions or rituals (e.g., needs things to be in a certain order, certain place, or certain color)	○	○	○	○	○
58. Avoids or is upset by visiting new places or meeting new people	○	○	○	○	○
59. Becomes easily upset with changes in routine, new activities, or surprises	○	○	○	○	○
60. Has difficulties with transition	○	○	○	○	○
61. Needs you to change your behavior to avoid becoming upset (e.g., needs you to avoid eating certain foods, needs you to avoid driving a certain way, needs you to say things a certain way)	○	○	○	○	○
62. Speaks in an unusual tone of voice **(Note: If your child does not use spoken language, please chose Never)**	○	○	○	○	○
63. Repeats meaningless sounds (e.g., says "Digadigadiga" for no reason)	○	○	○	○	○

© 2023 Boston Children's Hospital. All Rights Reserved.
For more information, contact the PROM Study Team: ASDPROM@childrens.harvard.edu.

Restricted and Repetitive Behaviors and Interests

To what extent does your child do each of the following:

	Never	Rarely	Sometimes	Often	Always
64. Echoes other people	○	○	○	○	○
65. Repeats phrases from TV and movies ("Language scripting") **(Note: If your child does not use spoken language, please chose Never)**	○	○	○	○	○
66. Perseverates or gets stuck on certain thoughts	○	○	○	○	○

Sensory Processes

To what extent does your child do each of the following:

	Never	Rarely	Sometimes	Often	Always
67. Peers at objects out of the corder of eyes	○	○	○	○	○
68. Craves deep pressure (e.g., tight hugs or weighted vests)	○	○	○	○	○
69. Is upset by certain noises (e.g., vacuum cleaners, hair dryers)	○	○	○	○	○
70. Puts things in mouth that are not food (e.g., toys, paint, dirt)	○	○	○	○	○
71. Avoids touching certain things (e.g., sand, grass)	○	○	○	○	○
72. Has a high tolerance for pain	○	○	○	○	○
73. Holds or pack food in mouth	○	○	○	○	○
74. Eats a limited variety of foods	○	○	○	○	○

© 2023 Boston Children's Hospital. All Rights Reserved.
For more information, contact the PROM Study Team: ASDPROM@childrens.harvard.edu.

Challenging Behaviors

To what extent does your child do each of the following:

	Never	Rarely	Sometimes	Often	Always
75. Is physically aggressive toward self (e.g., hits, bites, scratches self, bangs head)	○	○	○	○	○
76. Is physically aggressive toward others (e.g., hits, kicks, bites, scratches, pinches, pulls hair)	○	○	○	○	○
77. Expresses thoughts of wanting to hurt others	○	○	○	○	○
78. Destroys or breaks things when upset	○	○	○	○	○
79. Has temper tantrums or meltdowns	○	○	○	○	○
80. Interrupts when others are speaking	○	○	○	○	○

Mental Health

To what extent does your child do each of the following:

	Never	Rarely	Sometimes	Often	Always
81. Expresses thoughts of self-harm or suicide	○	○	○	○	○
82. Is a victim of bullying	○	○	○	○	○
83. Worries too much	○	○	○	○	○
84. Picks at skin or nails	○	○	○	○	○
85. Seems sad	○	○	○	○	○
86. Easily frustrated	○	○	○	○	○
87. Has sudden changes in mood	○	○	○	○	○
88. Sees things that are not there (Visual hallucinations)	○	○	○	○	○
89. Hears things that are not there (Auditory hallucinations)	○	○	○	○	○
90. Decreased or flattened emotions	○	○	○	○	○

© 2023 Boston Children's Hospital. All Rights Reserved.
For more information, contact the PROM Study Team: ASDPROM@childrens.harvard.edu.

Impulse/ADHD

To what extent does your child do each of the following:

	Never	Rarely	Sometimes	Often	Always
91. Runs away (e.g., bolts, wanders)	○	○	○	○	○
92. Is easily distracted; has difficulty paying attention	○	○	○	○	○
93. Is hyperactive (e.g., fidgety, restless)	○	○	○	○	○
94. Is impulsive; acts without thinking	○	○	○	○	○

Adaptive/Toileting Skills

To what extent does your child do each of the following:

	Never	Rarely	Sometimes	Often	Always
95. Is potty trained during the day	○	○	○	○	○
96. Is able to clean/wipe himself/ herself after going to the bathroom	○	○	○	○	○
97. Smears/plays with stool or urine	○	○	○	○	○
98. Toilets in appropriate places	○	○	○	○	○
99. Holds back stool	○	○	○	○	○
100. Is able to dress independently	○	○	○	○	○

© 2023 Boston Children's Hospital. All Rights Reserved.
